# Evaluation of a Bacterial Single-Cell Protein in Compound Diets for Rainbow Trout (*Oncorhynchus mykiss*) Fry as an Alternative Protein Source

**DOI:** 10.3390/ani10091676

**Published:** 2020-09-17

**Authors:** Abbas Zamani, Maryam Khajavi, Masoumeh Haghbin Nazarpak, Enric Gisbert

**Affiliations:** 1 Fisheries Department, Faculty of Natural Resources and Environment, Malayer University, 65719-95863 Malayer, Iran; marykhajavi@yahoo.com; 2 New Technologies Research Center, Amirkabir University of Technology, 15916-33311 Tehran, Iran; haghbin@aut.ac.ir; 3 IRTA, Centre de Sant Carles de la Rápita (IRTA-SCR), Aquaculture Program, Crta. del Poble Nou Km 5.5, 43540 Sant Carles de la Rápita, Spain; enric.gisbert@irta.cat

**Keywords:** amino acid profile, enzyme activity, fatty acid profile, growth performance, rainbow trout, single-cell protein

## Abstract

**Simple Summary:**

Fish meal (FM), as the main protein source, is used in aquafeeds due to its good nutritional profile and palatability. In recent years, because of the high cost and uncertainty in FM supply, studies have been focused to identify and evaluate alternative protein ingredients to minimize FM and reducethe cost of formulated feeds. Currently, plant protein ingredients and animal by-products are used as alternative protein sources to FM, but these components have some nutritional limitations, such as beingrich in anti-nutritional elements and deficient in certain essential amino acids. Among alternative protein sources, single-cell protein (SCP) such as bacteria, yeasts and microalgae, is considered a promising substitute for animal- or plant-derived ingredients. In this work, we aimed to evaluate the replacement of FM with a bacterial SCP, a by-product of the monosodium L-glutamic acid produced by microbial fermentation of vegetal raw materials, in diets for rainbow trout (*Oncorhynchus mykiss*) fry. Results indicated that the maximum replacement of FM by SCP in terms of growth and feed efficiency performance was up to 50%, while the broken-line regression analyses using DHA muscle content and weight gain showed that this value ranged between 46.9 to 52% SCP depending on the parameter considered.

**Abstract:**

A 60-day trial was conducted in rainbow trout (*Oncorhynchus mykiss*) fry (initial weight = 2.5 ± 0.6 g) to evaluate the potential use of a bacterial single-cell protein (SCP) as an alternative protein source. Five experimental diets with different levels of fishmeal replacement (0, 25, 50, 75 and 100%) and no amino acid supplementation were tested. At the end of the trial, we found that fry fed diets, replacing 25 and 50% of fishmeal with bacterial SCP, were 9.1 and 21.8% heavier, respectively, than those fed the control diet (*p* < 0.05), while Feed Conversion Ratio (FCR) values were also lower in comparison to the reference group. These results were also supported by Protein Efficiency Ratio (PER) and Lipid Efficiency Ratio (LER) values that improved in fish fed diets replacing 50% fishmeal by bacterial SCP. The inclusion of SCP enhanced Feed intake (FI) (*p* < 0.05), although FI was reduced at higher inclusion levels (>50%), which was associated to feed palatability. High levels of bacterial SCP (>50%) affected the muscular amino acid and fatty acid profiles, imbalances that were associated to their dietary content. The broken-line regression analysis using muscle DHA content and weight gain data showed that the maximum levels of fishmeal replacement by bacterial SCP were 46.9 and 52%, respectively.

## 1. Introduction

The rainbow trout (*Oncorhynchus mykiss*) is the most important cold-water cultivated fish species in Iran, of which theproduction has increased from 9000 t in 2010 to 718,736 t in 2018, placing this country as the world producer leader of this salmonid species [1]. Similarly, the worldwide production of this species has also increased, but to a lesser extent than in Iran, raising from 752,404 t in 2010 to 848,051.5 t in 2018, a production value that represents circa 2% of the total finfish production [1]. One of the most important aspects of the rainbow trout farming industry is its continuous attempt to increase production efficiency and its long-term sustainability, and in this sense, the improvement of feed formulation by screening new ingredients is necessary [2,3]. For many years, fishmeal (FM) has been used as the main protein source in formulated feeds for aquatic species, due to its good nutritional profile and palatability [4,5]. In recent years, because of the high cost and uncertainty in FM supply, continuous efforts have been invested in looking for alternative protein sources for aquafeeds. As Hua et al. [6] recently reviewed, plant protein ingredients (e.g., soybean, corn gluten and rapeseed meals) and animal by-products (e.g., meat and bone meals, poultry meal) are currently used as alternative protein sources to FM. Although plant-based proteins have been indicated to be important ingredients for aquafeeds, these components have some nutritional limitations, such as anti-nutritional elements, whereas the industry itself has limited potential to expand production without putting additional pressure on land, water, and phosphorous resources. Among alternative protein sources (e.g., fishery and aquaculture by-products, insects, food waste, macroalgae), microbial biomass also known as “microbial meal” or “single-cell protein” (SCP) is considered as a promising substitute for animal- or plant-derived ingredients [7,8,9,10,11]. Among microorganisms, bacteria, yeasts and microalgae are usually displayed to have the highest potential for aquafeeds, especially bacteria and yeasts that have a relatively high protein content (50–80% and 45–55%, respectively), and an amino acid profile comparable to FM. Additionally, they can potentially be used as either functional feed additives or as alternative raw materials [6]. Regardless of their contrasted nutritional value and applicability in aquafeed formulation [2,3,12,13,14,15], their use is still limited due to the high cost of production [6,16]. However, SCP suitability and inclusion rates need to be evaluated at a species-specific level, with a focus on their digestibility and nutrient bioavailability within the microbial biomass [16]. In this context, extensive feeding trials are also necessary for their validation under different rearing and husbandry conditions, especially at early life stages of development, when fish nutritional requirements are higher than at older ages.

Among different SCP sources, we focused on an SCP concentrate obtained from dried killed, non-genetic modified organisms (GMO) bacterial biomass. This is a by-product of monosodium L-glutamic acid production by means of microbial fermentation of vegetal raw materials from crop origin [17]. Nutritionally, this SCP is rich in highly digestible protein (68–72%) and contains high levels of several essential amino acids such aslysine, threonine, tryptophan, isoleucine and valine, which makes this bacterial SCP a very attractive and cost-effective ingredient for replacing FM in aquafeeds [18].

The aim of the present study was to evaluate the effects of bacterial SCP as a substitute for FM in rainbow trout fry diets in order to determine its maximal level of inclusion in terms of somatic growth, feed efficiency parameters, muscle proximate composition and functionality of the digestive system.

## 2. Materials and Methods

### 2.1. Fish, Experimental Design and Diets

Fry of rainbow trout were obtained from a commercial fish farm (Nahavand, Hamedan, Iran) and transported by road (1 h trip) to the Fisheries Laboratory of the Malayer University (Hamedan, Iran). Prior to the experiment, fish were acclimated in a 2000 L-tank for two weeks; during this period, fry were fed a commercial diet (crude protein 48%, crude lipid 14%, ash 9%, crude fiber 2% and moisture 6%, SFT1, SFT1, Faradaneh, Shahr-e Kord, Iran).During this period, fish were hand-fed twice a day at a feeding ration of 3% of their body weight (BW), which approached apparent satiation. Using a completely randomized design, three triplicate tanks were assigned to each of the five experimental diets. A total of 225 rainbow trout fry (initial BW: 2.51 ± 0.55 g, mean ± standard deviation) were distributed into 15 cylindroconical tanks (volume: 100 L; 15 fish per tank) connected to an open-flow water system. During the experiment (60 days), physical and chemical water parameters were monitored twice per day to maintain optimal quality conditions. The nutritional trial was conducted under a 12:12 h light:dark photoperiod, water temperature of 15.0 ± 1.5 °C and mean oxygen concentration of 9.6 ± 0.1 mg L^−1^ (WTW, Multi 3410, Weilheim, Germany). Water quality parameters (pH, ammonia and nitrites) were measured using Aquamerck test kits (Merck, Darmstadt, Germany); mean water pH values were 8.2 ± 0.1, whereas levels of ammonia and nitrites were <0.1 mg L^−1^. Initial feeding rate was 3% of the stocked biomass, whereas feeding rate was daily adjusted depending on the amount of uneaten feed pellets recovered from the bottom of the tank from the previous day. For this purpose, one hour after feed administration, uneaten pellets were recovered from the bottom of the tank, dried in an oven (100 °C) and their dry weight used for estimating the amount of uneaten feed and calculating fry feed intake for the following day.

Experimental diets were formulated to be isonitrogenous (45.6% crude protein), isolipidic (21.5% crude lipid) and isoenergetic (5.43 kcal g^−1^) and met the nutritional requirements for this species [19]. Graded levels (0, 25, 50, 75 and 100%) of bacterial SCP (PL68®, Intraco Ltd.,Antwerp, Belgium) were tested replacing equivalent quantities of FM. Diets were named according to FM replacement levels by bacterial SCP (D_SCP0_, D_SCP25_, D_SCP50_, D_SCP75_ and D_SCP100_). In addition, in order to ensurediets remained isoproteic, the inclusion of meat and bone meals was also decreased as levels of bacterial SCP increased (Table 1). The list of ingredients used for diet formulation and the proximate composition of manufactured diets are presented in Table 1. Experimental diets were prepared as described by Welker et al. [20] with slight modifications. In particular, feed ingredients were homogenously mixed and a dough was obtained for further extrusion. Pellets were extruded (MPT-E120 extruder, Iran). Pelleting conditions were as follows: 20 s at 120–150 °C and 30 bars of pressure (mesh size: 2 mm). Pellets were dried at 15 °C for 10 min in cooler after extrusion.

Animal experimentation procedures were approved by the Ethics Committee in the research of Hamadan University of Medical Sciences with the ID of IR.UMSHA.REC.1397.994.

### 2.2. Sampling Procedures and Measured Parameters

At the end of the feeding trail, all fish from each individual tank were fasted for 24 h and sacrificed with an overdose of clove oil in order to measure their BW (g) and total length (TL) to the nearest 0.1 g and 1 mm, respectively (FX2000i digital scale, Bradford, England). Growth and feed performance indicators were calculated using the following standard formulae:

- Weight Gain (WG, g) = final BW (g) − initial BW (g);
- Specific Growth Rate (SGR, % body weight/day) = 100 [(Ln final BW (g) − Ln initial BW(g)/time (days)];
- Condition Factor (K) = 100 × [BW (g)/TL^3^ (cm)];
- Feed intake (FI; % body weight/day) = [total dry mass intake/(initial body weight × final body weight)^0.5^/number of days fed]×100;
- Feed Conversion Ratio (FCR) = Consumed feed/WG;
- Survival rate (S, %) = (final fish number/initial fish number) × 100;
- Hepatosomatic Index (HSI, %) = [liver weight (g)/BW (g)] × 100;
- Lipid Efficiency Ratio (LER) = WG/total amount of lipid ingested;
- Protein Efficiency Ratio (PER) = WG/total amount of protein ingested

Once fish were measured at the end of the trial, eightfish from each tank were randomly selected and frozen at −80 °C for fillet proximate analysis and to determine amino acid and fatty acid composition. The remaining sevenfish from each tank were dissected in order to determine their HSI, as well as evaluate the activity of selected pancreatic digestive enzymes. For determination of digestive enzyme activities, fish were ventrally dissected on chilled trays, their digestive tracts excised and the adherent adipose and connective tissues removed. Then, the pyloric caeca (PC) and intestine were separated and immediately frozen in liquid nitrogen and stored at −80 °C until further analysis.

### 2.3. Proximate, FattyAcid and Amino Acid Analyses

The proximate composition of the experimental diets and fish muscle (50 g of sample) was determined using standard procedures described by the Association of Official Analytical Chemists (AOAC) [21]. Briefly, moisture content was obtained by weight loss after drying samples in an oven (Memmert Universal Oven, UN30) at 105 °C until they reached a constant weight. Protein was determined by measuring nitrogen, using the Kjeldahl (Kjeltec^TM^ 2300, Foss Tecator, Hoganas, Sweden) technique (N × 6.25). Total lipid was extracted by n-hexane using the Soxhlet method (Soxtec^TM^ 2050, Foss Tecator) and ash content was determined for each dried sample after incineration in a muffle furnace (Nabertherm model K, Nabertherm GmbH, Bremen, Germany) at 550 °C for 5 h. Feed energy content was calculated using gross energy values of 5.63 Kcal g^−1^ for crude protein, 9.43 Kcal g^−1^ for crude fat and 4.11 Kcal g^−1^ for carbohydrates [21].

Fatty acid (FA) analysis was performed in triplicate for each experimental diet and muscle samples. Total lipids from feed samples and fillets were extracted by homogenization in chloroform/methanol (2:1, *v*/*v*) containing 0.01% butylated hydroxy toluene (BHT) as antioxidant [22]. Fatty acid methyl esters (FAME) in samples were analyzed using a Philips PU 4400 gas chromatograph (Phillips Scientific, Cambridge, United Kingdom) equipped with a fused silica capillary column BPX-70 (30 m × 0.25 mm, film thickness of 0.22 μm) and a flame ionization detector. The carrier gas and split rate were helium and 1/100, respectively. The temperature program included a gradient from 140 up to 250 °C with an increase rate of 1.5 °C min^−1^. FAME levels were determined by comparison of their retention times with commercial standards (Sigma, St. Louis, MO, USA).

Amino acid (AA) composition for diet and fillet from each experimental replicate was conducted by hydrolysis with HCl (6N) at 110 °C for 24 h and performed the derivatization using o-phthaldialdehyde (OPA) prior to HPLC analysis [23]. The amino acid profile was obtained using a Kinetex, EVO C18 HPLC column (150 × 4.6 mm; particle size: 5 µm, using acetonitrile as solvent; Waters) at a flow rate of 1 mL min^−1^ with UV detection and applied *Chromulan* software for data analysis.

### 2.4. Activity of Pancreatic Digestive Enzymes

Frozen PC and intestines were partially thawed at 4 °C for 2 h. Then, tissues (dilution 1:20, *w*/*v*) from each tank replicate were pooled and homogenized in cold buffer (50 mM Tris-HCl buffer, pH 8.0 containing 10 mM CaCl_2_). Thereafter, the homogenate was centrifuged at 14,000× *g* for 45 min at 4 °C and the resultant supernatants were collected, and aliquots were stored at −80 °C until digestive enzyme analysis. For each enzyme activity, assay dilution tests were previously done to ensure optimum enzyme to substrate ratio [24]. There were less than 6 months between sampling time and enzyme activity measurements in order to avoid the potential loss of enzyme activities [25]. All enzyme activities were measured at room temperature (23.0 ± 0.2 °C) by means of a spectrophotometer (UV/VS Ultro Spec 2000, Pharmacia Biotech, LabX, Midland, ON, Canada). The specific assay conditions for each enzyme were as below.

Trypsin (EC 3.4.21.4) activity was determined using BAPNA (N-α-benzoyl-DL-arginine p-nitroanilide) as substrate in 50 mM Tris-HCl, 20 mM CaCl_2_ + 1.5 mM NaCl buffer (pH 8.2) according to the method of Erlanger et al. [26]. One unit of activity was defined as the enzyme releasing 1 μmol p-nitroaniline per minute at λ = 410 nm. Bile salt-activated lipase (EC 3.1.1.3) activity was measured by assessing the hydrolysis of ρ-nitrophenyl myristate dissolved in 100 mM Tris-HCl, 20 mM CaCl_2_ buffer (pH 8.0), 0.25 mM 2-methoxyethanol and 5 mM sodium cholate buffer [27]. One unit of enzyme activity was defined as 1μmol of ρ-nitrophenol released per minute at λ = 405 nm. Alpha-amylase (EC 3.2.1.1) activity was estimated according to the Bernfeld’s [28] procedure using starch as substrate (1 g soluble starch in 100 mL 20 mM sodium phosphate, 6 mM NaCl buffer, pH 6.9) and 3,5-dinitrosalicylic acid as the reagent. One unit of activity was defined as 1μmol of maltose released per minute and absorbance was measured at λ = 540 nm. Data were expressed as specific activity (U mg protein^−1^), and the concentration of soluble protein in extracts was determined by the method of Lowry et al. [29] using bovine serum albumin (0–1 mg ml^−1^) as a standard. Enzyme activities from all samples were analyzed in triplicate (methodological replicates).

### 2.5. Economic Profit Analysis

A cost analysis was performed in order to compare the cost of feed required to produce 1 kg of fish biomass considering the replacement of FM by bacterial SCP and feed efficiency values (FCR). The economic conversion ratio (ECR) and economic profit index (EPI) were calculated according to Martínez-Llorens et al. [30]:

- ECR (US$ kg^−1^) = feed cost (US$ kg^−1^) × feed conversion ratio (kg diet kg fish^−1^);
- EPI (US$ fish^−1^) = [final weight (kg fish^−1^) × fish sale price (US$ kg^−1^) − ECR (US$ kg^−1^) × weight increase (kg)].

### 2.6. Statistical Analyses

Data were presented as means ± standard deviation (SD), and a probability value of *p* < 0.05 was considered as significant. Following confirmation of normality and homogeneity of variance, an ANOVA was performed followed by the Duncan’s multiple range test when statistically significant differences were detected among experimental groups. Data expressed as percentages were arcsine-transformed prior to the ANOVA analysis. Statistical analyses were performed using the SPSS (Version 21.0, SPSS Inc., Chicago, IL, USA). Broken-line regression method was used to determine the breakpoint that represents the maximum replacement of fishmeal by bacterial SCP in diets for rainbow trout fry based on WG values and docosahexaenoic acid (DHA) muscle content. These analyses were conducted using SigmaPlot for Windows version 12 (Systat Software Inc.,Regus House, Slough, Berkshire, UK).

## 3. Results

### 3.1. Fatty Acid and Amino Acid Composition of Experimental Diets

The FA composition of experimental diets is shown in Table 2. Replacement of FM with bacterial SCP significantly changed the FA profiles of evaluated diets (*p* < 0.05). The control diet (D_0SCP_) contained the highest levels of HUFA, especially EPA and DHA (4.2% and 10.5%, respectively), and the lowest levels of total n-6 PUFA (14.4%). In contrast, the D_100SCP_ diet had the highest levels of n-6 PUFA (18.9%), especially linoleic acid (18.9%) and the lowest levels of total n-3 HUFA (13.4%), n-3/n-6 ratio (0.77%), as well as the lowest DHA/EPA ratio (2.2) among experimental diets. Dietary levels of n-6 PUFA gradually increased with increasing rates of FM replacement with bacterial SCP, whereas total n-3 HUFA decreased (*p* < 0.05). In all experimental diets, the most abundant saturated fatty acids (SFA), mono-unsaturated fatty acids (MUFA), poly-unsaturated fatty acids (PUFA) and highly-unsaturated fatty acids (HUFA) were C16:0, C18:1n-9, C18:2n-6 and C22:6n-3, respectively.

The amino acid composition of experimental diets is presented in Table 3. The levels of the following amino acids, serine, asparagine + alanine, threonine, histidine, cysteine + methionine, lysine, tyrosine and argininesignificantly decreased with increasing levels of bacterial SCP in diets (*p* < 0.05). In contrast, the levels of aspartate + glutamate, proline, valine and isoleucine significantly increased with increasing bacterial SCP levels in experimental diets (*p* < 0.05).

### 3.2. Survival, Growth Performance, Feed Efficiency and Body Condition Parameters

**Figure 1:**
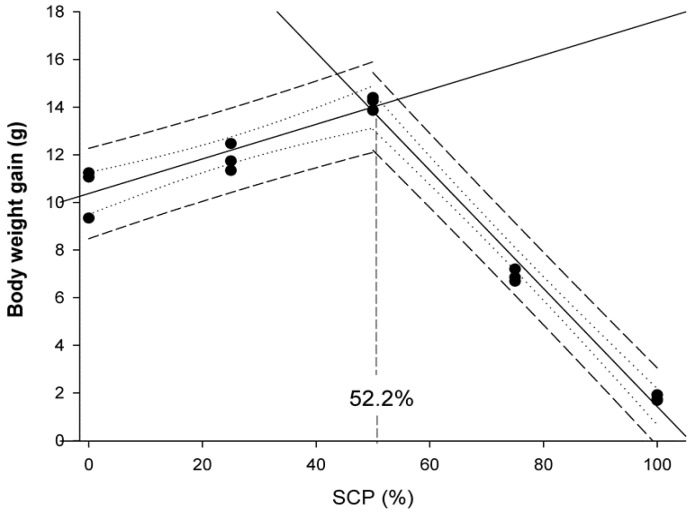
Broken-line analysis of weight gain (WG) in rainbow trout (*O. mykiss*) fry fed experimental diets containing graded levels of bacterial single cell protein (SCP) replacing fishmeal during a 60-day feeding trial. The dashed line showsthe prediction interval, whereas the dotted line represented the confidence interval. Both intervals were established at 95% of confidence.

No differences in survival rates (S = 100%) were observed among dietary groups (Table 4; *p* > 0.05). Regarding growth performance, somatic growth in rainbow trout fry was significantly affected by experimental diets (Table 4; *p* < 0.05). In particular, the highest WG and SGR values were recorded in fish fed the D_SCP50_ diet, while the lowest ones were observed in fry fed the D_100SCP_ diet (*p* < 0.05); the other dietary groups showed intermediate values. When considering WG data, the broken-line regression analysis revealed that the maximal dietary level of bacterial SCP replacing FM in diets for rainbow trout fry was 52.2% (Figure 1).

Dietary SCP significantly affected feed performance; in particular, the lowest and highest FCR values were found in fry fed D_50SCP_ and D_100SCP_ diets, respectively, whereas the other dietary groups showed intermediate values (Table 4; *p* < 0.05). In addition, FI values in fry fed D_100SCP_ and D_75SCP_ diets were lower than those recorded in fish from D_0SCP_, D_25SCP_ and D_50SCP_ groups (*p* < 0.05). The condition factor was significantly lower in fry fed D_100SCP_, D_75SCP_ and D_0SCP_ diets compared to those fed D_25SCP_ and D_50SCP_ diets (*p* < 0.05). There were no statistically significant differences in HSI values among experimental groups (Table 4; *p* > 0.05). The highest LER and PER values were found in fry fed the D_50SCP_ diet, whereas the lowest ones were observed in fish from the D_100SCP_ group (Table 4; *p* < 0.05).

### 3.3. Proximate Composition, Fatty acid and Amino Acid Muscle Profiles

The proximate composition of the muscle of rainbow trout fry was significantly affected by experimental diets (Table 5). Similar values in the crude protein content of muscle were found in fry fed D_0SCP_, D_25SCP_, D_50SCP_ and D_75SCP_ diets; these values were significantly higher than those in the D_100SCP_ group (*p* < 0.05). The lowest crude lipid muscular levels were recorded in fry fed D_75SCP_ and D_100SCP_ diets (*p* < 0.05), whereas no differences were found among the other dietary groups. In addition, moisture muscle levels of fry fed D_75SCP_ and D_100SCP_ diets were lower than those of the other groups (*p* < 0.05). Ash contents were highest in fry fed D_75SCP_ and D_100SCP_ diets, whereas the lowest values were found in fry fed the D_50SCP_ diet (*p* < 0.05).

**Figure 2:**
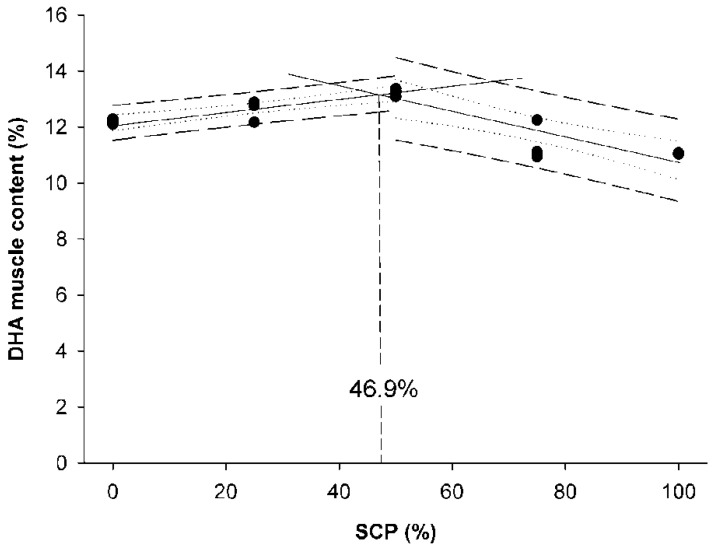
Broken-line analysis of DHA muscle content in rainbow trout (*O. mykiss*) fry fed experimental diets containing graded levels of bacterial single cell protein (SCP) replacing fishmeal during a 60-day feeding trial. The dashed line showsthe prediction interval, whereas the dotted line represented the confidence interval. Both intervals were established at 95% of confidence.

The FA composition of the muscle in rainbow trout fry fed experimental diets was affected by the replacement of FM by bacterial SCP in diets (Table 6). In particular, levels of SFAs differed among dietary groups; thus, fry fed D_100SCP_ and D_25SCP_ diets showed the lowest and the highest SFAs content, respectively (*p* < 0.05). Palmitic (C16:0) and stearic (C18:0) acids were the most abundant SFA in the muscle of fry in all experimental groups. MUFAs content was lower in fish fed the D_100SCP_ diet in comparison to the control diet (D_0SCP_), whereas the other dietary groups showed intermediate values (*p* < 0.05). The most abundant MUFA was the oleic acid (C18:1n-9), whereas its lowest content was found in the fillet of fry fed the D_100SCP_ diet (*p* < 0.05). PUFA levels significantly increased with increasing levels of FM replacement by bacterial SCP, with maximal values found in the muscle of fry form the D_100SCP_ group (*p* < 0.05). The highest content in linolenic acid (C18:3n-6) was found in fish fed the D_50SCP_ diet, while the lowest value was found in fish fed the D_100SCP_ (*p* < 0.05). The highest and the lowest levels of linoleic acid (C18:2n-6) were found in fish fed D_100SCP_ and D_0SCP_ diets, respectively. The levels of HUFAs increased with increasing levels of FM replacement by bacterial SCP up to 50% of FM replacement (D_0SCP_, D_25SCP_ and D_50SCP_), and then decreased in the rest of experimental groups (*p* < 0.05). The amount of total fatty acids of the n-3 series in the muscle of fry increased from D_0SCP_ to D_50SCP_ groups, whereas it decreased in fry fed D_75SCP_and D_100SCP_diets (*p* < 0.05). In addition, fry fed the D_100SCP_ diet contained the highest concentration of total fatty acids from the n-6 series in their muscular tissue. In this sense, the highest EPA, DHA and EPA/DHA levels were found in fish fed D_0SCP_, D_25SCP_ and D_50SCP_ diets, whereas the lowest values were found in D_75SCP_ and D_100SCP_ groups (*p* < 0.05). Arachidonic acid (C20:4n-6) levels found in the muscle of fry fed D_25SCP_ and D_50SCP_ were higher than in the rest of dietary groups (*p* < 0.05).The maximum FM replacement by bacterial SCP without impairing DHA muscle content was found at 46.9% as results from the broken-line analysis shownin Figure 2.

The amino acid profile of the muscle of rainbow trout fed diets in which FM was replaced by bacterial SCP is presented in Table 7. Levels of aspartate + glutamate, leucine and isoleucine in the muscle of fry fed D_25SCP_, D_50SCP_, D_75SCP_ and D_100SCP_ diets were significantly higher than those in fry fed the D_0SCP_ diet (*p* < 0.05). Muscle proline content in fish fed D_50SCP_, D_75SCP_ and D_100SCP_ diets was significantly higher than in D_0SCP_ and D_25SCP_ groups (*p* < 0.05). Valine, glycine, serine, histidine and lysine levels in fry fed D_75SCP_ and D_100SCP_ diets were significantly higher than in the other dietary groups (*p* < 0.05). The levels of threonine, cysteine + methionineand tyrosine in the muscle of fry fed D_0SCP_ and D_25SCP_ diets were significantly higher than in the other groups (*p* < 0.05). Fry fed the control diet (D_0SCP_) had the higher asparagine + alanine and arginine levels in muscle than in the other nutritional groups (*p* < 0.05).

### 3.4. Activity of Pancreatic Digestive Enzymes

Specific activities of the pancreatic digestive enzymes trypsin, bile salt-activated lipase and α-amylase are shown in Table 8. The replacement of FM by bacterial SCP affected the activity of the assessed pancreatic digestive enzymes, regardless of the region of the digestive tract considered (*p* < 0.05). In particular, trypsin activity in the PC was significantly higher in the D_50SCP_ diet in comparison to D_75SCP_ and D_100SCP_ diets. Similarly, the highest trypsin specific activities in the intestinal region were found in fish fed theD_50SCP_ diet (*p* < 0.05). The specific activity of bile salt-activated lipase from PC was similar to that reported from the intestine. Regarding the specific activity of bile salt-activated lipase, values progressively increased from D_0SCP_ to D_50SCP_ groups without significant differences (*p* > 0.05), while activity values were significantly decreased in fish fed D_75SCP_ and D_100SCP_ diets (*p* < 0.05). The activity of α-amylase in the PC was significantly higher in fish fedtheD_50SCP_ diet in comparison to those fed other diets. Regarding the activity of α-amylase in the intestine, the highest activity values were found in fry fed the D_50SCP_ diet, whereas no differences were recorded in fish fed D_0SCP_, D_25SCP_ and D_50SCP_ diets. Fry fed D_75SCP_ and D_100SCP_ diets showed lower activity values in comparison to above-mentioned groups.

### 3.5. Economic Profit Analysis

Values of the economic conversion ratio and economic profit index were significantly affected by the level of FM replacement by bacterial SCP (Table 9; *p* < 0.05) The lowest and highest ECR values were found in D_50SCP_ and D_100SCP_ diets, respectively, whereas the other dietary groups showed intermediate values (*p* < 0.05). In addition, EPI values calculated for the D_50SCP_ diet was significantly higher than those from the other tested diets (*p* < 0.05). The lowest EPI values were recorded in fish fed the D_100SCP_ diet (*p* < 0.05).

## 4. Discussion

Research on the potential use of different SCP sources in aquafeeds started in the late 1970s [12,13,31,32], and because of the encouraging results obtained, research is still ongoing in this field [6]. The results of the present study indicated that bacterial SCP obtained as a by-product of the monosodium L-glutamic acid production can replace up to 52% of the FM in compound diets containing 46% protein for rainbow trout fry without negatively affecting their growth and feed efficiency performances. These findings confirmed its nutritional value as an alternative protein source when included in compound diets. In particular, fry fed D_SCP25_ and D_SCP50_ diets were 9.1 and 21.8% heavier than those fed the control diet, respectively. These results may be attributed to the good and balanced nutritional profile of the tested bacterial SCP that substantially improved growth performance parameters (BWf, SGR, WG, K), similar to results reported in other salmonid species testing different bacterial SCP sources. For instance, Overland et al. [33] found that SCP from *Methylococcus capsulatus* could be included in diets for Atlantic salmon (*Salmo salar*)—up to 52% of the dietary protein with no adverse growth effects, whereas the same authors found that this source of SCP could only be included in rainbow trout diets up to 38%. Similarly, SCP from *Methylobacterium extorquens* could replace 55% of FM in Atlantic salmon diets without negatively affecting growth performance [11], whereas it could replace up to 10% of soybean meal in rainbow trout diets [3]. Other studies have reported that in Atlantic salmon, the amount of bacterial protein meals in diets can be up to 36% FM, results that were associated to an improvement of growth performance and feed efficiency in comparison to thecontrol diet [7]. In the present study, growth performance results were in agreement with their respective FCR values, although FCR in fry fed the D_50SCP_ diet were better than in those fed the control diet (D_SCP0_), as well as in fry fed diets with a moderate inclusion of SCP (D_25SCP_). In contrast, the replacement of FM at higher levels than 50% with bacterial SCP reduced feed efficiency parameters. These results may be attributed to the lower palatability of diets, as fish offered D_75SCP_ and D_100SCP_ diets did not swallow these feeds as their congeners from the other groups did. In particular, fry split pellets from D_75SCP_ and D_100SCP_ diets after being tasted in the oral cavity, whereas in other cases, pellets were completely ignored by fish. This issue may also have affected the activity of the assessed pancreatic enzymes in pyloric caeca and in the anterior intestine, since digestive enzyme activity depends on FI among other factors [34]. Other studies in salmonids evaluating different bacterial meal sources have also reported a reduction in FI and an effect in FCR; however, the level of FM replacement by bacterial SCP in compound diets that reduced FI and negatively affected FCR values varied depending on the study considered and the source of bacterial SCP considered [2,7,8,35]. Similarly, different fish husbandry conditions, feed formulation and nutrient composition may substantially affect FCR values in research.

Different studies have found contradictory results in terms of the effect of different bacterial SCP meals on FI in fish. For instance, Rumsey et al. [36] suggested that dietary free purines in SCP sources negatively affected feed palatability; thus, reducing feed intake. Similarly, Kiessling and Askbrandt [35] and Hardy et al. [3] found that a reduction in FI in rainbow trout fed with high levels of bacterial SCP could be counteracted by adding palatability-enhancing ingredients such as betaine. In contrast, other authors found that the inclusion of bacterial SCP meal produced with methane as a carbon source did not affect appetite in Atlantic salmon nor in rainbow trout [7,8,37,38]. The above-mentioned different results in terms of FI and FCR reported by several authors may be attributed to the nutritional profile, palatability, digestibility of the bacterial protein meal used, as well as the process by which this ingredient was processed and dried for dietary applications [16].

Diet quality and digestibility were also influenced by the inclusion of bacterial SCP as LER and PER values indicated. In particular, rainbow trout fry fed the D_50SCP_ diet showed the best values in LER and PER, showing the good quality of the tested diet in terms of protein and lipid contents [19]. These results were also supported by the higher activity of trypsin in the anterior intestine in comparison to the control group and other dietary treatments. Trypsin secretion in the gut may be promoted by the dietary amino acid and peptide profiles [39]. However, differences in LER values may not be attributed to differences in bile salt-activated lipase, since activity values did not differ among fry fed the control and D_25SCP_ and D_50SCP_ diets. Thus, differences in LER values between the control and D_50SCP_ may be related to the dietary fatty acid profile and lipid classes [40]. The replacement of FM at >50% by bacterial SCP might also have affected diet digestibility as other authors reported; however, under present experimental conditions, this reduction in pancreatic enzyme activities in rainbow trout fed D_75SCP_ and D_100SCP_ diets could be also due to the above-mentionedreduction in FI. These results were similar to those reported by other authors that found that different levels of bacterial SCP inclusion impaired diet digestibility. For instance, Perera et al. [2] found that the dietary inclusion of bacterial meal at 17% in rainbow trout diets reduced PER values and increased nitrogen excretion, whereas it lowered protein digestibility. Similarly, the replacement of 55% FM by SCP from *M. extorquens* in diets for Atlantic salmon negatively affected diet digestibility [11]. These diverse findings may be attributed to the lower digestibility of cell walls [41], the high content in nucleic acids of SCP meals [36,42,43] and poorer efficiency in nutrient absorption [44,45,46], among other factors [16].

In this study, the proximate composition of the muscle in rainbow trout fry fed D_75SCP_ and D_100SCP_ diets was affected, showing lower protein and lipid contents. Considering that the tested diets were isoproteic and isolipidic, such differences in muscle proximate composition may be attributed to the lower growth performance observed in the above-mentioned groups. Similar results were reported for red drum (*Sciaenops ocellatus*) [47], hybrid striped bass (*Morone chrysops* × *M. saxatilis*) [48] and rainbow trout [3,49] when FM was replaced by SCPs. Regarding the AA profile of tested diets, all diets replacing FM with bacterial SCP meal met the nutritional requirements of rainbow trout fry considering the recommendations provided by the NRC [19] for this particular stage of development. Under present experimental conditions, the AA composition of the muscle in rainbow trout fry closely matched the AA composition of experimental diets containing graded levels of bacterial SCP. In particular, lower levels of essential AAs, such as cysteine, methionine, arginine, histidine, lysine and threonine, were found in fry fed the D_100SCP_ diet. Similar results were obtained in Atlantic salmon [7] and whiteleg shrimp (*Litopenaeus vannamei*) [5] fed diets containing bacterial meal from *Corynebacterium ammoniagenes*. This could indicate that the AA requirements of fish were not met by the higher level of bacterial SCP in the diet, as evidenced by fish growth reduction. Although diets theoretically met the nutritional requirements of rainbow trout fry, the low levels of the above-mentioned AAs in fry musculature may be associated to problems in diet digestibility due to the presence of bacterial cell walls in SCP meals [41]. Problems in digestibility of diets containing high levels of bacterial SCP may impair AA absorption and generate imbalances in AA composition that may also promote their oxidation [50,51] and limit their availability [2]. Although diet digestibility was not addressed in our study, these results may indicate a reduction in diet digestibility at higher levels of FM replacement by bacterial SCP; thus, further studies need to be conducted to test this hypothesis. Another potential hypothesis that may explain the above-mentioned results is the presence of free dietary AAs in diets that may be absorbed faster thanother AAs supplied as polypeptides or intact proteins, which may also potentially generate a time-based AA imbalance [52]; thus, increasing the magnitude of AA imbalance in diets containing the highest levels of bacterial SCP. Therefore, the present data indicated that the dietary supplementation of the above-mentioned essential AAs might be advisable when replacing FM at higher levels than 50% in compound diets (46% protein) for rainbow trout fry. However, AA supplementation need to be accompanied by technological solutions focused on increasing SCP digestibility (i.e., cell wall disruption) [16]. The final choice of high replacement of FM by bacterial SCP will be set up on a cost-benefit basis, considering the balance between the cost of dietary AA supplementation and the improvement of bacterial meal digestibility and the improvement of fish performance and quality indicators.

The inclusion of bacterial SCP in rainbow trout fry diets affected body lipid content and the FA profile of the muscular tissue. In general terms, the FA profile of the muscle of rainbow trout fry fed graded bacterial SCP levels clearly reflected the FA composition of the diet. The replacement of FM with higher levels of bacterial SCP (>50%)resulted in a reduction in SFA, MUFA, n-3 PUFA and n-3 HUFA contents in fry muscle, values that were strongly influenced by their respective dietary levels. High levels of 18:2n-6 (LA) were observed in the muscle of rainbow trout fry fedthe D_100SCP_ diet, whereas lower levels in several essential fatty acids, such asC18:3n-3 (LNA), C20:5n-3 (EPA), C22:6n-3 (DHA) and C20:4n-6 (ARA), were found in this dietary group. Linolenic acid is considered an essential fatty acid for salmonid species since it serves as a substrate for biosynthesis of EPA and DHA [53].Thus, the lower levels of these fatty acids might also explain the lower performance of fry fed diets containing higher levels of bacterial SCP (D_75SCP_ and D_100SCP_) [35]. However, higher muscular levels of LA, the precursor of AA, were not associated to an increase in ARA in fry from D_75SCP_ and D_100SCP_, results that may be attributed to a limited capacity of bioconversion of LA into ARA at this particular stage of development [54].

Finally, the effect of FM replacement by bacterial SCP on feed cost production was also calculated. The lowest ECR and highest EPI values were obtained on fish fedthe D_50SCP_ diet. The best FCR was obtained with theD_50SCP_ diet, thereby lowering final cost of production compared to the control diet. Calculated ECR in fish fedthe D_50SCP_ diet would represent a saving of US$ 0.32 kg^−1^ (17.5%) compared to the control diet (US$ 1.83 kg^−1^).

## 5. Conclusions

The present study indicated that bacterial SCP, obtained as a by-product of monosodium L-glutamic acid production by means of microbial fermentation of vegetal raw materials, could replace up to 52% of FM in compound diets for rainbow trout fry as indicated bygrowth performance data, whereas this replacement was only of 46.9% when muscle DHA content was considered. In particular, fry fedthe D_50SCP_ diet showed a better performance than those from the control group. These results were also supported by PER and LER values, which were also improved in fish fed diets containing 50% bacterial SCP, which indicated that this source of bacterial SCP may be a nutritionally balanced ingredient when used at moderate doses at early life stages. The inclusion of this alternative protein ingredient enhanced FI and reduced FCR values, although FI was reduced and FCR increased at inclusion levels higher than 50%, which was associated to feed palatability issues when this bacterial SCP was used at high levels. Economically, our data indicated that the up to 50% replacement of fishmeal with bacterial SCP in diets containing 46% protein may be viable using the protein source tested in this work, as the lowest ECR and highest EPI values were recorded in fish fed the D_50SCP_ diet. In addition, high levels of bacterial SCP (>50%) affected the muscular AA and FA profiles in fry, imbalances that were associated to their dietary content. Although formulated diets were balanced in terms of essential AA, at the end of the trial we found lower levels of cysteine, methionine, arginine, histidine, lysine andthreonine found in fry fed the D_100SCP_ diet, which was associated to digestibility problems when FM was completely replaced by bacterial SCP.

## Figures and Tables

**Table 1:** Feed ingredient list and proximate composition of experimental diets.

Ingredients (%)	Experimental Diets ^a^				
	D_0SCP_	D_25SCP_	D_50SCP_	D_75SCP_	D_100SCP_
Fish meal (62% crude protein) ^b^	50	37.5	25	12.5	0
PL68^®^^c^	0	12.5	25	37.5	50
Soybean meal (45% crude protein) ^d^	6.48	6.48	6.48	6.48	6.48
Empyreal^®^ 75 ^e^	4	4	4	4	4
Meat and bone meal (52.8% crude protein) ^f^	6	4.94	3.88	2.82	1.76
Corn gluten meal	9	8.02	7.04	6.06	5.08
Wheat flour	13	13.84	14.78	15.52	16.36
Fish oil ^g^	7	8.2	9.3	10	10
Soybean oil	2	2	2	2.6	3.8
Mineral premix ^h^	1	1	1	1	1
Vitamin premix ^i^	1	1	1	1	1
BHT ^j^	0.02	0.02	0.02	0.02	0.02
Binder ^k^	0.5	0.5	0.5	0.5	0.5
Total	100	100	100	100	100
Proximate composition (%)					
Dry matter	95.50	95.57	95.05	95.12	94.94
Crude protein	45.68	45.72	45.47	45.51	45.44
Crude Lipid	21.50	21.48	21.46	21.45	21.44
Crude fiber	1.20	1.28	1.39	1.45	1.53
Ash	8.02	7.91	7.87	7.73	7.68
NFE ^l^	19.10	19.18	18.86	18.98	18.85
Energy (Kcal/g) ^m^	5.44	5.45	5.43	5.44	5.43

^a^ Diet abbreviations: D_0SCP_ (control diet, no fish meal replacement by bacterial single cell protein—SCP), D_25SCP_, D_50SCP_, D_75SCP_ and D_100SCP_, diets containing 25, 50, 75 and 100% of bacterial SCP replacing fish meal at similar values, respectively. ^b^ Parskilka (Mazandaran, Iran). The lipid content of fishmeal was circa 7%. ^c^ Non-GMO bacterial SCP concentrate based on microbial fermentation of vegetal raw materials from crop origin (Intraco Ltd., Belgium). Composition: protein 68%, fat 3.25%, crude fiber 1%, ash 10% and moisture 10%. Amino acid profile: lysine, 2.35%; methionine + cysteine, 1.15%; isoleucine, 2.45%; tryptophan, 0.60%; threonine, 3.0%; arginine, 2.95%; valine, 3.25%; alanine, 5.02 %; glutamic acid, 18.1%. ^d^ Khavardasht Co. (Gorgan, Golestan, Iran). ^e^ Corn-based protein concentrate (Cargill, Inc.). Composition: protein, 76.2%; fat, 4.5%; carbohydrates, 5.3%; crude fiber, 1%; ash, 1.3%; moisture, 8.5%; energetic content, 3.35 Kcal g^−1^. ^f^ Gohar Daneh Shargh Co. (Mashhad, Iran) obtained from cattle. ^g^ Anchovy oil (Havorash: Boshehr, Iran). Fatty acid composition (%): C14:0, 0.05; C15:0, 0.7; C16:0, 20.6; C17:0, 0.9; C18:0, 3.9; Σn-3, 21.24; Σn-6, 1.88. ^h^ Mineral premix (mg kg^−1^): KCl, 200; KI, 60); COCl_2_ 6H_2_O, 7; CuSO_4_ 5H_2_O, 14; FeSO_4_ H_2_O, 400; ZnSO_4_ H_2_O, 200; MnSO_4_ H_2_O, 80; Na_2_SeO_3_ 5H_2_O, 65; MgSO_4_·7H_2_O, 3000; Ca(H_2_PO_4_) H_2_O, 20,000; NaCl, 136; Zeolit, 5840; career up to 1kg. ^i^ Vitamin premix (U kg^−1^): vitamin B1, 12,000 mg; vitamin B2, 5000 mg; vitamin B3, 35,000 mg; vitamin B5, 30,000 mg; vitamin B6, 6000 mg; B7, 60 mg; vitamin B9, 2000 mg; vitamin B12, 50 mg; vitamin A, 80,000,000 IU; vitamin D3, 200,000,000 IU; vitamin E, 44,000 IU; vitamin K3, 5000 mg; vitamin C, 500,000 mg; inositol, 100,000 mg; antioxidant (*Ethoxyquin)*, 150,000 mg, career up to 1 kg. ^j^ Antioxidant: Butylated hydroxy toluene (Garmab Shimi, Iran). ^k^ Antifungal agent: Natural hydrated sodium calcium aluminum silicates. ^l^ Nitrogen-free extract. ^m^ Calculated on the basis of 5.63, 9.43 and 4.11 Kcal g^−1^ of protein, fat and carbohydrate, respectively [19].

**Table 2:** Fatty acid composition expressed as a percentage of total fatty acids of experimental diets containing graded levels of bacterial single cell protein (SCP) replacing fishmeal used for feeding rainbow trout (*Oncorhynchus mykiss*) fry for 60 days *.

Fatty Acids	Experimental Diets				
	D_0SCP_	D_25SCP_	D_50SCP_	D_75SCP_	D_100SCP_
C14:0	3.29 ± 0.05 ^c^	3.03 ± 0.08 ^b^	2.94 ± 0.08 ^b,c^	2.78 ± 0.02 ^b^	2.56 ± 0.06 ^a^
C15:0	0.59 ± 0.08	0.42 ± 0.07	0.66 ± 0.05	0.66 ± 0.09	0.68 ± 0.03
C16:0	20.37 ± 0.13 ^c^	20.54 ± 0.07 ^c^	20.56 ± 0.02 ^c^	19.66 ± 0.08 ^b^	18.36 ± 0.16 ^a^
C17:0	0.69 ± 0.02 ^b^	0.76 ± 0.09 ^b^	0.67 ± 0.01 ^b^	0.61 ± 0.01 ^a^	0.51 ± 0.01 ^a^
C18:0	5.67 ± 0.06 ^d^	5.63 ± 0.03 ^d^	5.47 ± 0.02 ^c^	5.02 ± 0.03 ^b^	4.43 ± 0.03 ^a^
C20:0	0.33 ± 0.01 ^b^	0.34 ± 0.02 ^b^	0.35 ± 0.01 ^b^	0.34 ± 0.02 ^b^	0.25 ± 0.02 ^a^
C22:0	0.09 ± 0.01 ^a^	0.27 ± 0.02 ^b^	0.24 ± 0.01 ^b^	0.27 ± 0.04 ^b^	0.22 ± 0.02 ^b^
C24:0	0.11 ± 0.01	0.13 ± 0.02	0.10 ± 0.03	0.14 ± 0.01	0.08 ± 0.02
Σ SFAs	31.16 ± 014 ^c^	31.15 ± 0.18 ^c^	31.01 ± 0.19 ^c^	29.48 ± 0.21 ^b^	27.11 ± 0.14 ^a^
C16:1n-7	4.54 ± 0.15 ^b^	4.92 ± 0.11 ^c^	4.71 ± 0.01 ^b,c^	4.56 ± 0.07 ^b^	4.29 ± 0.04 ^a^
C17: 1n-7	0.42 ± 0.04	0.65 ± 0.07	0.61 ± 0.01	0.54 ± 0.07	0.52 ± 0.01
C18:1n-9	29.57 ± 0.13 ^a^	29.83 ± 0.31 ^a^	31.06 ± 0.16 ^b^	31.56 ± 0.24 ^b^	31.43 ± 0.09 ^b^
C20:1	1.19 ± 0.07	1.25 ± 0.04	1.25 ± 0.02	1.28 ± 0.01	1.25 ± 0.04
Σ MUFAs	35.72 ± 0.32 ^a^	36.66 ± 0.10 ^b^	37.64 ± 0.15 ^c^	37.96 ± 0.06 ^c^	37.53 ± 0.03 ^c^
C18:2 n-6	14.40 ± 0.09 ^c^	13.31 ± 0.12 ^b^	12.83 ± 0.09 ^a^	14.20 ± 0.25 ^c^	18.86 ± 0.16 ^d^
C18:3 n-6	0.04 ± 0.00 ^a^	0.09 ± 0.01 ^b^	0.09 ± 0.02 ^b^	0.14 ± 0.02 ^b^	0.07 ± 0.01 ^c^
C18:3 n-3	1.61 ± 0.01 ^b^	1.44 ± 0.01 ^a^	1.49 ± 0.02 ^a^	1.64 ± 0.10 ^b^	1.91 ± 0.05 ^c^
Σ PUFAs	16.01 ± 0.09 ^b^	14.84 ± 0.10 ^a^	14.42 ± 0.03 ^a^	15.98 ± 0.33 ^b^	20.82 ± 0.20 ^c^
C20:3 n-3	0.16 ± 0.01 ^a^	0.24 ± 0.01 ^c^	0.20 ± 0.01 ^b^	0.19 ± 0.01 ^b^	0.14 ± 0.01 ^a^
C20:4 n-6	0.71 ± 0.01 ^c^	0.60 ± 0.08 ^c^	0.48 ± 0.03 ^b^	0.43 ± 0.04 ^b^	0.25 ± 0.06 ^a^
C20:5 n-3	4.19 ± 0.02	4.25 ± 0.06	4.24 ± 0.01	4.28 ± 0.09	4.07 ± 0.07
C22:4 n-6	0.61 ± 0.07 ^b^	0.71 ± 0.04 ^b^	0.64 ± 0.01 ^b^	0.62 ± 0.02 ^b^	0.36 ± 0.01 ^a^
C22:5 n-6	0.34 ± 0.03 ^a^	0.47 ± 0.06 ^b^	0.51 ± 0.01 ^b^	0.49 ± 0.02 ^b^	0.36 ± 0.02 ^a^
C22:5 n-3	0.49 ± 0.01 ^c^	0.46 ± 0.01 ^c^	0.43 ± 0.04 ^b^	0.39 ± 0.01 ^b^	0.28 ± 0.01 ^a^
C22:6 n-3	10.45 ± 0.13 ^c^	10.37 ± 0.08 ^c^	10.21 ± 0.09 ^c^	9.91 ± 0.08 ^b^	8.88 ± 0.06 ^a^
Σ HUFAs	16.95 ± 0.55 ^b^	17.10 ± 0.59 ^b^	16.72 ± 0.08 ^b^	16.31 ± 0.39 ^b^	14.36 ± 0.39 ^a^
Σ n-3	16.90 ± 0.17 ^b^	16.76 ± 0.21 ^a,b^	16.57 ± 0.01 ^a,b^	16.41 ± 0.07 ^a,b^	15.29 ± 0.12 ^a^
Σ n-6	16.06 ± 0.01 ^c^	15.18 ± 0.08 ^a^	14.56 ± 0.10 ^b^	15.87 ± 0.24 ^c^	19.89 ± 0.01 ^d^
n-3/n-6	1.05 ± 0.01 ^b^	1.10 ± 0.02 ^c^	1.14 ± 0.01 ^c^	1.03 ± 0.02 ^b^	0.77 ± 0.01 ^a^
EPA/DHA	0.40 ± 0.01 ^a^	0.41 ± 0.01 ^a^	0.42 ± 0.01 ^a^	0.43 ± 0.01 ^a,b^	0.45 ± 0.01 ^b^
PUFAs/SFAs	0.51 ± 0.01 ^b^	0.47 ± 0.01 ^a^	0.46 ± 0.01 ^a^	0.54 ± 0.02 ^c^	0.76 ± 0.02 ^d^
AA/EPA	0.16 ± 0.01 ^c^	0.14 ± 0.02 ^c,b^	0.11 ± 0.01 ^b^	0.10 ± 0.01 ^b^	0.06 ± 0.02 ^a^

* Data are reported as mean ± SD (n = 3). ^a,b,c,d^ Means with different superscript letterswithin each row are significantly different (*p* < 0.05). Abbreviations: C14:0 (myristic acid), C15:0 (pentadecylic acid), C16:0 (palmitic acid), C17:0 (margaric acid), C18:0 (stearic acid), C20:0 (arachidic acid), C22:0 (behenic acid), C24:0 (lignoceric acid), C16:1n-7 (palmitoleic acid), C17:1n-7 (heptadecanoic acid), C18:1n-9 (elaidic acid), C20:1n-9 (gondoic acid) C18:2 n-6 (linoleic acid), C18:3 n-6 (linolenic (GLA) acid), C18:3 n-3 (linolenic (ALA) acid), C20:3 n-3 (eicosatrienoic acid), C20:4 n-6(arachidonic acid; AA), C20:5 n-3 (eicosapentaenoic acid; EPA), C22:4 n-6 (docosatetraenoic acid), C22:5 n-6 (osbond acid),C22:5 n-3 (docosapentaenoic acid), C22:6 n-3 (docosahexaenoic acid; DHA), SFAs (saturated fatty acids), MUFAs (monounsaturated fatty acids), PUFAs (poly-unsaturated fatty acids), HUFAs (highly-unsaturated fatty acids). For diet abbreviations, see Table 1.

**Table 3:** Amino acid composition expressed as a percentage of the total amino acids of experimental diets containing graded levels of bacterial single cell protein (SCP) replacing fishmeal used for feeding rainbow trout (*O. mykiss*) fry for 60 days *.

Amino Acid	Experimental Diets				
	D_0SCP_	D_25SCP_	D_50SCP_	D_75SCP_	D_100SCP_
Asp + Glu	22.98 ± 0.13 ^a^	27.91 ± 0.43 ^b^	32.81 ± 0.56 ^c^	35.84 ± 0.94 ^c^	37.78 ± 1.14 ^d^
Gly	12.03 ± 1.28	11.84 ± 0.60	11.34 ± 0.32	11.23 ± 0.65	10.97 ± 0.93
Ser	13.27 ± 0.42 ^b^	12.94 ± 0.10 ^b^	12.61 ± 0.68 ^b^	12.12 ± 0.57 ^b^	11.58 ± 0.02 ^a^
Asn + Ala	6.58 ± 0.38 ^c^	4.81 ± 0.22 ^b^	3.69 ± 0.26 ^a^	3.54 ± 0.41 ^a^	3.24 ± 0.31 ^a^
Thr	7.68 ± 0.43 ^c^	6.97 ± 0.14 ^c^	5.09 ± 0.26 ^b^	4.73 ± 0.31 ^b^	3.98 ± 0.24 ^a^
His	6.71 ± 0.23 ^b^	6.44 ± 0.43 ^b^	5.69 ± 0.31 ^b^	5.04 ± 0.15 ^a^	4.79 ± 0.27 ^a^
Pro	2.77 ± 0.15 ^a^	2.88 ± 0.32 ^a^	2.96 ± 0.13 ^a^	3.28 ± 0.57 ^b^	3.50 ± 0.18 ^c^
Val	2.52 ± 0.61 ^a^	3.18 ± 0.24 ^b^	3.22 ± 0.11 ^b^	3.58 ± 0.38 ^b^	3.90 ± 0.21 ^c^
Cys + Met	5.88 ± 011 ^b^	5.57 ± 0.21 ^b^	5.30 ± 0.11 ^b^	3.91 ± 0.17 ^a^	3.78 ± 0.23 ^a^
Lys	4.06 ± 0.98 ^b^	2.42 ± 0.28 ^a^	2.26 ± 0.28 ^a^	2.04 ± 0.07 ^a^	1.93 ± 0.27 ^a^
Tyr	1.30 ± 0.20 ^b^	1.13 ± 0.14 ^b^	1.06 ± 0.07 ^b^	0.56 ± 0.17 ^a^	0.39 ± 0.11 ^a^
Arg	5.92 ± 0.22 ^c^	5.41 ± 0.43 ^c^	4.68 ± 0.41 ^b^	4.38 ± 0.49 ^b^	3.97 ± 0.16 ^a^
Ile	5.58 ± 0.41 ^a^	6.37 ± 0.21 ^b^	6.59 ± 0.46 ^b^	6.87 ± 0.27 ^b^	7.21 ± 0.15 ^b^
Leu	1.98 ± 0.43	2.11 ± 0.43	2.16 ± 0.06	2.17 ± 0.03	2.47 ± 0.40

* Data are reported as mean ± SD (n = 3). ^a,b,c,d^ Means with different superscript letters in each row are significantly different (*p* < 0.05). Abbreviations: Asp (aspartic acid), Glu (glutamic acid), Gly (glycine), Ser (serine), Asn (asparagine), Ala (alanine), Thr (threonine), His (histidine), Pro (proline), Val (valine), Cys (cysteine), Met (methionine), Lys (lysine), Tyr (tyrosine), Arg (arginine), Ile (isoleucine), Leu (leucine). For diet abbreviations, see Table 1.

**Table 4:** Growth parameters of rainbow trout (*O. mykiss*) fry fed experimental diets containing graded levels of bacterial single cell protein (SCP) replacing fishmeal for 60 days *.

Parameters	Experimental Diets				
	D_0SCP_	D_25SCP_	D_50SCP_	D_75SCP_	D_100SCP_
Initial BW (g)	2.51 ± 0.55	2.51 ± 0.55	2.51 ± 0.55	2.51 ± 0.55	2.51 ± 0.55
Final BW (g)	13.05 ± 1.04 ^c^	14.36 ± 0.57 ^d^	16.68 ± 0.28 ^e^	9.42 ± 0.26 ^b^	4.29 ± 0.12 ^a^
WG (g)	10.55 ± 1.04 ^c^	11.85 ± 0.57 ^d^	14.17± 0.28 ^e^	6.91± 0.26 ^b^	1.78± 0.12 ^a^
SGR (% BW day^−1^)	2.74 ± 0.13 ^c^	2.90 ± 0.06 ^d^	3.15 ± 0.02 ^e^	2.20 ± 0.04 ^b^	0.89 ± 0.04 ^a^
FCR	0.96 ± 0.01 ^b^	0.89 ± 0.04 ^b^	0.76 ± 0.01 ^a^	1.11 ± 0.05 ^c^	2.07 ± 0.27 ^d^
Survival (%)	100	100	100	100	100
FI (% BW day^−1^)	2.61 ± 0.28 ^b^	2.94 ± 0.05 ^c^	3.14 ± 0.13 ^c^	2.56 ± 0.12 ^b^	1.72 ± 0.08 ^a^
K factor	0.94 ± 0.03 ^b^	0.99 ± 0.06 ^c^	1.05 ± 0.03 ^c^	0.92 ± 0.02 ^a,b^	0.87 ± 0.01 ^a^
HSI (%)	1.40 ± 0.12	1.42 ± 0.13	1.42 ± 0.14	1.49 ± 0.11	1.31± 0.08
LER	4.84 ± 0.07 ^c^	5.23 ± 0.24 ^c^	6.13 ± 0.08 ^d^	4.20 ± 0.19 ^b^	2.25 ± 0.28 ^a^
*PER*	2.28 ± 0.04 ^c^	2.45 ± 0.11 ^c^	2.89 ± 0.04 ^d^	1.97 ± 0.09 ^b^	1.07 ± 0.13 ^a^

* Data are mean ± SD (n = 3). ^a,b,c,d^ Means without a common superscript letter in each row are significantly different (*p* < 0.05). For diet abbreviations, see Table 1.

**Table 5:** Muscle proximate composition in rainbow trout (*O. mykiss*) fry fed experimental diets containing graded levels of bacterial single cell protein (SCP) replacing fishmeal for 60 days *.

Parameters	Experimental Diets				
	D_0SCP_	D_25SCP_	D_50SCP_	D_75SCP_	D_100SCP_
Moisture	75.65 ± 0.21 ^a^	75.34 ± 0.19 ^a^	75.26 ± 0.28 ^a^	76.48 ± 0.30 ^b^	77.61 ± 0.15 ^b^
Crude protein	17.40 ± 1.13 ^b^	17.56 ± 0.61 ^b^	17.61 ± 0.24 ^b^	17.04 ± 0.40 ^b^	16.68 ± 0.29 ^a^
Crude lipid	5.64± 0.69 ^c^	5.81 ± 0.19 ^c^	5.92± 0.24 ^c^	5.15 ± 0.11 ^b^	4.14± 0.32 ^a^
Ash	1.31± 0.04 ^b^	1.29± 0.05 ^b^	1.21± 0.03 ^a^	1.33± 0.02 ^c^	1.37± 0.02 ^c^

* Data are reported as mean ± SD (n = 3). ^a,b,c^ Means with different superscript letter in each row are significantly different (*p* < 0.05). For diet abbreviations, see Table 1.

**Table 6:** Fatty acid composition (% of total fatty acids) of rainbow trout (*O. mykiss*) fry muscle fed. Fish were fed experimental diets containing graded levels of bacterial single cell protein (SCP) replacing fishmeal for 60 days *.

Fatty Acid	Experimental Diets					
	Initial	D_0SCP_	D_25SCP_	D_50SCP_	D_75SCP_	D_100SCP_
C14:0	2.61 ± 0.01 ^a^	2.81 ± 0.09 ^c^	2.71 ± 0.04 ^c^	2.41 ± 0.11 ^c^	2.03 ± 0.08 ^b^	1.98 ± 0.04 ^b^
C15:0	0.45 ± 0.01 ^a^	0.52 ± 0.04 ^b^	0.60 ± 0.09 ^b^	0.51 ± 0.10 ^b^	0.29 ± 0.03 ^a^	0.35 ± 0.02 ^a^
C16:0	19.88 ± 1.21 ^a^	21.77 ± 1.08 ^c^	22.61 ± 0.21 ^c^	18.97 ± 0.34 ^b^	18.21 ± 0.70 ^b^	16.78 ± 0.88 ^a^
C17:0	0.54 ± 0.03 ^b^	0.65 ± 0.06 ^c^	0.61 ± 0.04 ^c^	0.57 ± 0.02 ^b^	0.52 ± 0.02 ^b^	0.41 ± 0.04 ^a^
C18:0	5.79 ± 0.52 ^a^	6.36 ± 0.45 ^b^	6.19 ± 0.30 ^b^	5.16 ± 0.06 ^a^	4.98 ± 0.30 ^a^	4.51 ± 0.21 ^a^
C20:0	0.21 ± 0.11 ^d^	0.23 ± 0.02 ^c^	0.22 ± 0.01 ^c^	0.20 ± 0.01 ^c^	0.17 ± 0.03 ^b^	0.10 ± 0.04 ^a^
C22:0	0.10 ± 0.01 ^c^	0.11 ± 0.01 ^b^	0.10 ± 0.01 ^b^	0.09 ± 0.04 ^a,b^	0.08 ± 0.01 ^a^	0.07 ± 0.01 ^a^
C24:0	0.05± 0.06 ^b^	0.06 ± 0.01 ^b^	0.05 ± 0.01 ^b^	0.05 ± 0.01 ^b^	0.04 ± 0.01 ^a^	0.03 ± 0.01 ^a^
Σ SFAs	29.63 ± 0.98 ^b,c^	32.51 ± 1.37 ^c^	33.09 ± 0.68 ^c^	27.96 ± 0.75 ^b^	26.32 ±0.65 ^b^	24.23 ± 0.97 ^a^
C16:1n-7	1.22 ± 0.12 ^a^	4.93 ± 0.09 ^c^	4.89 ± 0.18 ^c^	4.65 ± 0.16 ^c^	4.25 ± 0.14 ^b^	4.06 ± 0.08 ^b^
C17: 1n-7	0.20 ± 0.01 ^a^	0.63 ± 0.09 ^e^	0.62 ± 0.03 ^e^	0.53 ± 0.04 ^c^	0.46 ± 0.01 ^b^	0.31 ± 0.02 ^b^
C18:1n-9	39.21 ± 1.89 ^b^	35.60 ± 0.15 ^b^	35.53 ± 0.41 ^b^	34.34 ± 0.57 ^b^	33.53 ± 0.57 ^b^	31.01 ± 0.09 ^a^
Σ MUFAs	40.63 ± 1.13 ^c^	41.16 ± 0.75 ^c^	41.04 ± 1.14 ^c^	39.52 ± 0.62 ^b^	38.24 ± 1.10 ^b^	35.38 ± 0.87 ^a^
C18:2n-6	12.31 ± 0.71 ^a^	11.65 ± 0.16 ^a^	12.47 ± 0.09 ^a^	14.25 ± 0.81 ^b^	15.53 ± 0.45 ^b^	16.76 ± 0.31 ^b^
C18:3n-6	0.12 ± 0.09 ^a^	0.11 ± 0.03 ^a^	0.14 ± 0.04 ^a,b^	0.23 ± 0.01 ^b^	0.25 ± 0.03 ^b^	0.33 ± 0.04 ^d^
C18:3n-3	0.84 ± 0.10 ^b^	0.82 ± 0.07 ^b^	0.94 ± 0.01 ^b^	0.99 ± 0.05 ^b^	0.72 ± 0.07 ^a^	0.67 ± 0.06 ^a^
Σ PUFAs	13.27 ± 0.53 ^a^	12.58 ± 0.42 ^a^	13.55 ± 0.57 ^a^	15.47 ± 0.43 ^b^	16.50 ±0.81 ^b^	17.76 ± 0.24 ^c^
C20:3n-3	0.76 ± 0.16 ^b^	0.69 ± 0.09 ^b^	0.74 ± 0.02 ^b^	0.75 ± 0.05 ^b^	0.65 ± 0.03 ^a^	0.55 ± 0.04 ^a^
C20:4n-6	0.60 ± 0.07 ^b^	0.52 ± 0.03 ^a^	0.66 ± 0.05 ^b^	0.71 ± 0.06 ^b^	0.46 ± 0.08 ^a^	0.45 ± 0.01 ^a^
C20:5n-3	2.17 ± 0.04 ^b^	1.91 ± 0.07 ^b^	2.13 ± 0.16 ^b^	2.18 ± 0.14 ^b^	1.78 ± 0.12 ^a^	1.74 ± 0.08 ^a^
C22:4n-6	0.68 ± 0.14 ^b^	0.52 ± 0.06 ^b^	0.57 ± 0.13 ^b^	0.59 ± 0.12 ^b^	0.41 ± 0.02 ^a^	0.39 ± 0.04 ^a^
C22:5 n-6	0.42 ± 0.03 ^c^	0.35 ± 0.04 ^b^	0.36 ± 0.02 ^b^	0.37 ± 0.05 ^b^	0.33 ± 0.04 ^b^	0.24 ± 0.01 ^a^
C22:5 n-3	0.65 ± 0.09 ^b^	0.59 ± 0.01 ^b^	0.61 ± 0.04 ^b^	0.74 ± 0.10 ^b^	0.43 ± 0.08 ^a^	0.42 ± 0.02 ^a^
C22:6 n-3	12.33 ±0.21 ^b^	12.19 ± 1.38 ^b^	12.61 ± 0.38 ^b^	13.25 ± 0.14 ^b^	11.43 ± 0.72 ^a^	11.06 ± 0.02 ^a^
Σ HUFAs	17.61 ±1.12 ^b^	16.57 ± 0.98 ^b^	17.68 ± 0.88 ^b^	18.59 ± 1.19 ^b^	15.49 ± 0.71 ^a^	14.85 ± 0.59 ^a^
Σ n-3	16.75 ±1.11 ^b^	16.20 ± 1.12 ^b^	17.03 ± 0.95 ^b^	17.91 ± 0.35 ^b^	15.01 ± 0.39 ^a^	14.44 ± 0.63 ^a^
Σ n-6	14.13 ± 1.08 ^a^	13.15 ± 0.91 ^a^	14.20 ± 1.19 ^a^	16.15 ± 1.10 ^b^	16.98 ± 1.78 ^b^	18.17 ± 2.07 ^b^
n-3/n-6	1.18 ± 0.01 ^b^	1.23 ± 0.07 ^b^	1.20 ± 0.08 ^b^	1.11 ± 0.03 ^b^	0.88 ± 0.02 ^a^	0.79 ± 0.04 ^a^
EPA/DHA	0.18 ± 0.02 ^a^	0.16 ± 0.01 ^b^	0.17 ± 0.01 ^b^	0.16 ± 0.01 ^b^	0.15 ± 0.07 ^a^	0.15 ± 0.01 ^a^
PUFA/SFA	0.45 ± 0.03 ^a^	0.39 ± 0.02 ^a^	0.41 ± 0.03 ^a^	0.55 ± 0.04 ^b^	0.63 ± 0.04 ^b,c^	0.73 ± 0.08 ^c^
AA/EPA	0.28 ± 0.01 ^a^	0.27 ± 0.02 ^a^	0.31 ± 0.03 ^a,b^	0.33 ± 0.05 ^b^	0.26 ± 0.04 ^a^	0.26 ± 0.03 ^a^

* Data are reported as mean ± SD (n = 3). ^a,b,c,d,e^ Means with different superscript letter in each row are significantly different (*p* < 0.05). For diet and fatty acid abbreviations, see Table 1 and Table 2, respectively.

**Table 7:** Amino acid composition (% of total amino acids) of the muscle of rainbow trout (*O. mykiss*) fry fed experimental diets with graded levels of bacterial single cell protein (SCP) replacing fishmeal for 60 days *.

Amino Acid	Experimental Diets					
	Initial	D_0SCP_	D_25SCP_	D_50SCP_	D_75SCP_	D_100SCP_
Asp + Glu	20.57 ± 1.04 ^a^	20.70 ± 1.14 ^a^	23.37 ± 0.94 ^b^	25.53 ± 0.53 ^c^	27.25 ± 0.89 ^c^	27.66 ± 0.43 ^c^
Gly	13.69 ± 0.20 ^c^	13.80 ± 0.26 ^c^	12.71 ± 0.33 ^c^	11.96 ± 0.17 ^b^	11.13 ± 0.37 ^a^	10.71 ± 0.04 ^a^
Ser	11.97 ± 0.31 ^b^	12.07 ± 0.52 ^b^	11.26 ± 0.55 ^b^	11.17 ± 0.79 ^b^	10.02 ± 0.66 ^a^	8.54 ± 1.27 ^a^
Asn + Ala	6.18 ± 0.29 ^b^	6.22 ± 0.41 ^b^	5.70 ± 0.91 ^a^	5.31 ± 0.70 ^a^	5.29 ± 0.67 ^a^	5.21 ± 0.83 ^a^
Thr	11.68 ± 0.95 ^c^	11.71 ± 1.31 ^c^	10.85 ± 1.05 ^c^	9.51 ± 1.14 ^b^	8.46 ± 1.71 ^b^	7.11 ± 1.26 ^a^
His	3.69 ± 0.65 ^b^	3.81 ± 0.88 ^b^	3.54 ± 0.35 ^b^	3.26 ± 0.66 ^b^	2.49 ± 0.43 ^a^	2.45 ± 0.76 ^a^
Pro	6.84 ± 0.12 ^a^	6.90 ± 0.03 ^a^	7.55 ± 1.68 ^a^	8.89 ± 0.54 ^b^	9.21 ± 1.15 ^b^	11.53 ± 2.03 ^c^
Val	4.28 ± 0.15 ^a^	4.36 ± 0.26 ^a^	4.52 ± 0.74 ^a^	4.77 ± 1.24 ^a^	6.31 ± 0.28 ^b^	8.66 ± 1.23 ^b^
Cys + Met	5.97 ± 0.33 ^c^	6.03 ± 0.41 ^c^	5.41 ± 031 ^c^	4.08 ± 0.32 ^b^	3.74 ± 0.07 ^b^	2.74 ± 0.16 ^a^
Lys	4.74 ± 0.16 ^b^	4.85 ± 0.31 ^b^	4.53 ± 0.45 ^b^	4.25 ± 0.25 ^b^	3.01 ± 0.21 ^a^	2.83 ± 0.28 ^a^
Tyr	1.31 ± 0.05 ^b^	1.39 ± 0.11 ^b^	1.18 ± 0.04 ^b^	0.98 ± 0.03 ^a^	0.96 ± 0.32 ^a^	0.87 ± 0.36 ^a^
Arg	4.08 ± 0.53 ^c^	4.13 ± 0.78 ^c^	3.63 ± 0.48 ^b^	3.53 ± 0.61 ^b^	3.35 ± 0.96 ^b^	2.31 ± 0.29 ^a^
Ile	1.87 ± 0.11 ^a^	1.95 ± 0.15 ^a^	2.91 ± 0.26 ^b^	3.37 ± 0.20 ^b^	3.52 ± 0.45 ^b^	4.43 ± 0.18 ^b^
Leu	1.89 ± 0.10 ^a^	1.91 ± 0.03 ^a^	2.74 ± 0.75 ^b^	2.91 ± 0.51 ^b^	4.24 ± 0.98 ^c^	4.81 ± 0.81 ^c^

* Data are reported as mean ± SD (n = 3). ^a,b,c^ Means with different superscript letter in each row are significantly different (*p* < 0.05). For diet and amino acid abbreviations, see Table 1 and Table 3, respectively.

**Table 8:** Specific activity of trypsin, bile salt-activated lipase and α-amylase from the pyloric caeca and intestine of rainbow trout (*O. mykiss*) fed with graded levels of bacterial single cell protein (SCP) for 60 days *.

SCP Level (%)	Digestive Enzyme Activity (mU mg Protein^−1^)					
	Trypsin		Bile Salt-Activated Lipase		α-Amylase	
	Pyloric Caeca	Intestine	Pyloric Caeca	Intestine	Pyloric Caeca	Intestine
**D_0SCP_**	52.9 ± 1.7 ^c^	10.8 ± 0.4 ^c^	51.6 ± 0.7 ^c^	34.2 ± 0.2 ^c^	539 ± 9 ^b^	384 ± 16 ^b^
**D_25SCP_**	53.0 ± 2.8 ^c^	14.9 ± 0.3 ^d^	52.0 ± 1.3 ^c^	34.3 ± 1.4 ^c^	576 ± 10 ^b^	393 ± 18 ^b^
**D_50SCP_**	55.8 ± 3.3 ^c^	16.0 ± 0.7 ^e^	53.5 ± 0.9 ^c^	35.8 ± 1.0 ^c^	984 ± 55 ^c^	400 ± 12 ^b^
**D_75SCP_**	44.9 ± 2.8 ^b^	5.9 ± 0.3 ^b^	49.2 ± 1.1 ^b^	32.3 ± 0.7 ^b^	459 ± 12 ^a^	334 ± 13 ^a^
**D_100SCP_**	37.1 ± 1.8 ^a^	4.3 ± 0.4 ^a^	36.0 ± 1.0 ^a^	29.9 ± 0.7 ^a^	421± 17 ^a^	327 ± 15 ^a^

* Data are reported as mean ± SD (n = 3). ^a,b,c,d,e^ Means with different superscript letters within each column are significantly different (*p* < 0.05). For diet abbreviations, see Table 1.

**Table 9:** Economic profit analysis for the experimental diets formulated for rainbow trout (*O. mykiss*) fry replacing different levels of fishmeal with bacterial SCP.

Parameters	Experimental Diets				
	D_0SCP_	D_25SCP_	D_50SCP_	D_75SCP_	D_100SCP_
Feed cost (US$ kg^−1^)	1.99	1.97	1.95	1.93	1.90
ECR	1.83 ± 0.03 ^a^	1.76 ± 0.08 ^a^	1.51 ± 0.02 ^a^	2.29 ± 0.12 ^b^	3.93± 0.52 ^c^
EPI	0.04 ± 0.003 ^c^	0.05 ± 0.002 ^d^	0.06 ± 0.001 ^e^	0.03 ± 0.001 ^b^	0.01± 0.001 ^a^

* Data are mean ± SD (n = 3). ^a,b,c,d,e^ Means without a common superscript letter within each row are significantly different (*p* < 0.05). Feed cost was calculated using the price of ingredients from Iranian ingredient suppliers and from the bacterial SCP supplier (fishmeal, 2.3 US$ kg^−1^; fish oil, 1.7 US$ kg^−1^; bacterial SCP, 2.2 US$ kg^−1^). For diet abbreviations, see Table 1.
